# Monro-Kellie 4.0: moving from intracranial pressure to intracranial dynamics

**DOI:** 10.1186/s13054-025-05476-7

**Published:** 2025-06-05

**Authors:** Sérgio Brasil, Gustavo C. Patriota, Daniel Agustín Godoy, Jorge L. Paranhos, Andrés Mariano Rubiano, Wellingson S. Paiva

**Affiliations:** 1 Experimental Surgery Laboratory, Division of Neurological Surgery, University of São Paulo Medical School, São Paulo, Brazil; 2 Department of Neurosurgery, Hospital de Emergência e Trauma Senador Humberto Lucena, João Pessoa, Brazil; 3 Neurointensive Care, Sanatório Pasteur, Catamarca, Argentina; 4 Intensive Care and Neuroemergency, Santa Casa de Misericórdia, São João del Rei, Brazil; 5 Neurosciences and Neurosurgery, Universidad El Bosque, Bogotá, Colombia

**Keywords:** Intracranial compartmental syndrome, Intracranial compliance, Intracranial pressure, Acute brain injury, Traumatic brain injury

## Abstract

The Monro-Kellie doctrine, introduced in the late 18th century, was a groundbreaking concept aimed at explaining the interactions between intracranial volume components. It has since become a cornerstone of brain physiology, now recognized as intracranial dynamics. Initially, the doctrine focused on physiological observations of the three incompressible components of the cranial vault: brain tissue, blood, and cerebrospinal fluid (CSF). Over the centuries, advancements in neuroscience and medical technology have deepened our understanding of intracranial pressure (ICP) regulation, its pathophysiological implications and its role in neurological disorders. This revisitation of the Monro-Kellie doctrine examines how impairments in cerebrovascular autoregulation, brain compartmentalization and the glymphatic system interact in severely brain-injured patients, calling for new management strategies when facing these critical situations. Additionally, it reinforces the need for a holistic monitoring approach to improve early diagnostics and intervention. The evolution of ICP assessment has significantly shaped the management of brain trauma, spontaneous bleeding, ischemic stroke, and hydrocephalus. With the introduction of innovative tools such as brain ultrasound, automated pupillometry and noninvasive pressure waveform monitoring, ICP management is shifting toward more accessible and continuous evaluation strategies. This review explores how blending historical principles with cutting-edge innovations is transforming neuromonitoring and enhancing patient outcomes in critical care.

## A doctrine in constant evolution

The Monro-Kellie Doctrine (MK), formulated over 240 years ago, remains a fundamental framework for understanding intracranial pressure (ICP) regulation. It describes the brain, blood, and cerebrospinal fluid (CSF) as components confined within the fixed-volume compartment of the cranium. The doctrine was shaped by the scientific contributions of Alexander Monro, George Kellie, George Burrows, and Harvey Cushing, whose pioneering work laid the foundation for modern ICP management. From Monro’s anatomical observations to Cushing’s clinical breakthroughs, each played a critical role in refining this concept, now referred to as MK 1.0: “Any increase in the volume of one component must be compensated by a proportional reduction in another to maintain stable ICP” [1–6].

Over time, advancements in cerebral physiology have prompted significant revisions to the original Monro-Kellie Doctrine. It has become increasingly clear that the interaction between intracranial components is dynamic rather than static. In particular, venous dynamics play a crucial role in ICP regulation. Unlike arterial blood flow, which is primarily driven by systemic circulation, venous drainage from the brain is influenced by external factors such as increased intra-abdominal or intrathoracic pressure and mechanical compression of the jugular veins. These factors can cause venous congestion, raising ICP even in the absence of mass lesions or overt pathological processes. This realization led to the refinement of the doctrine into MK 2.0, which recognizes that venous occlusion or impaired outflow can elevate ICP independently of changes in arterial or CSF dynamics [7–13].

The concept of MK 3.0 emerged from clinical observations in conditions such as normal pressure hydrocephalus (NPH) and idiopathic intracranial hypertension (IIH). These cases revealed that mechanical changes within brain tissue can cause cerebral morphological changes even in the absence of mass lesions or radiological signs of elevated ICP [14]. Such morphological changes include, in particular, the distension of periventricular fibers in cases of NPH and optic nerve injury in cases of IIH [14]. On the other hand, in low grade communicating hydrocephalus, the brain can undergo structural adaptations in response to CSF leaks, leading to skull remodeling and reduction in brain volume due to interstitial fluid (IF) loss [9, 15–17]. These evolutionary perspectives for the concept are summarized in Table 1.

**Table 1:** Evolutionary perspectives on the Monro-Kellie doctrine

Monro-Kellie 1783-20 th century	The total volume within the rigid skull—comprising brain tissue, blood and CSF—is constant, so an increase in one component must be offset by a decrease in another to maintain normal ICP
Monro-Kellie 2.0 2016	ICP is no longer viewed in isolation but as part of a dynamic, interconnected system influenced by extracranial pressures, particularly from the thoracic and abdominal cavities. These compartments are anatomically and physiologically linked to the cranial vault through venous and cerebrospinal pathways
Monro-Kellie 3.0 2019	Changes in the elastic properties of the brain were seen in IIH and NPH patients, as remodeling of brain shape with minor brain shift in the skull vault, but CSF displacement
Monro-Kellie 4.0	Represents the paradigm shift from the traditional intracranial hypertension model to one centered on impaired intracranial dynamics in acute brain injuries, highlighting the need for a multimodal approach to ensure accurate assessment

CSF: cerebrospinal fluid, ICP: intracranial pressure, IIH: idiopathic intracranial hypertension, NPH: normal pressure hydrocephalus

### Monro Kellie doctrine 4.0

**Fig. 1:**
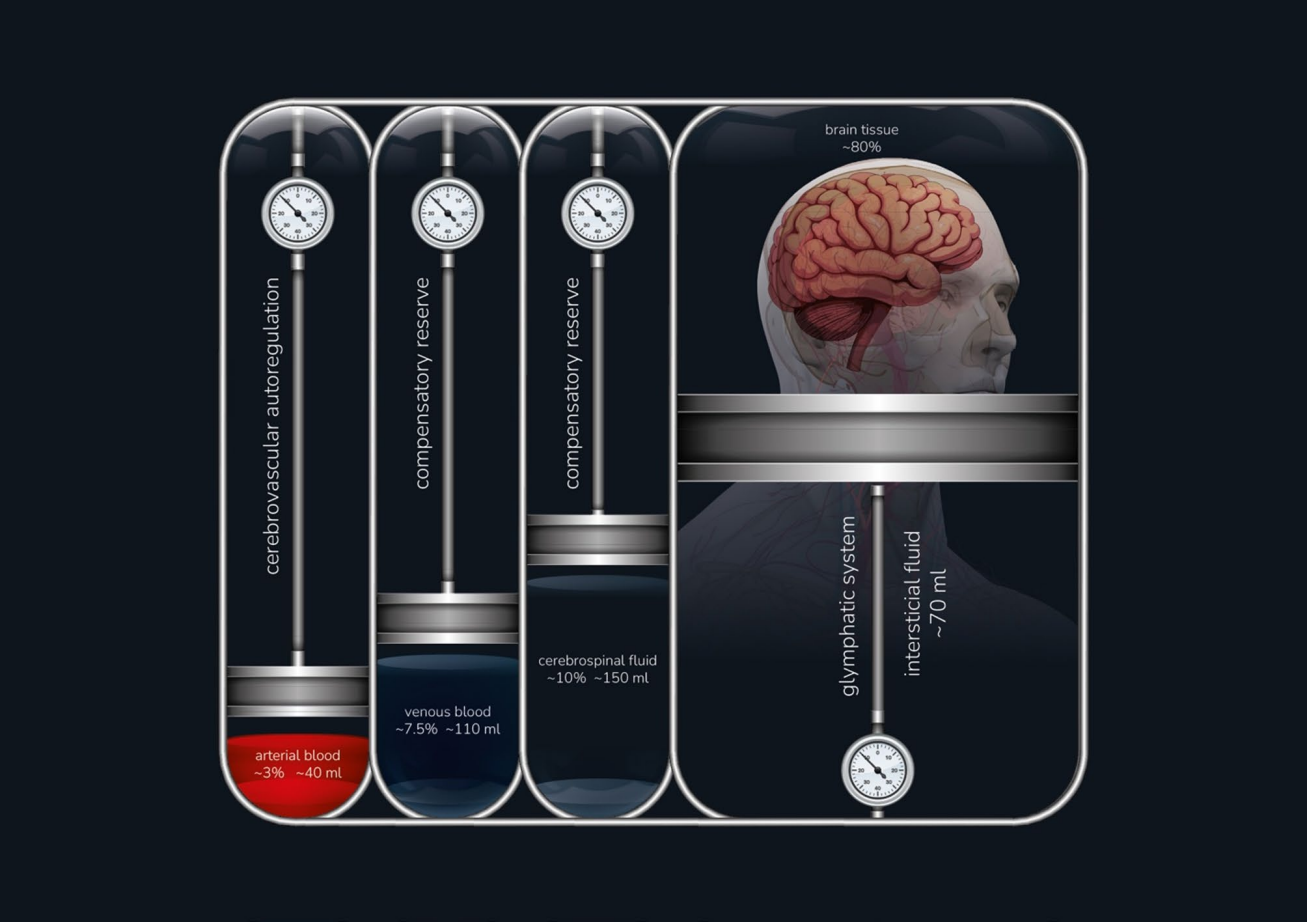
MONRO-KELLIE 4.0, the dynamic nature of intracranial components with their determinants for variation. The cerebrovascular autoregulation for arterial blood, the buffering reserve for venous and CSF volumes and the glymphatic activity to regulate interstitial space and consequently the brain tissue volume

From a clinical perspective, MK 4.0 represents a paradigm shift in the understanding and management of ICP in neurocritical care. It challenges the traditional view that ICP elevation is primarily driven by intracranial mass lesions, as proposed in MK 1.0, and highlights the broader role of cerebrovascular dynamics (Fig. 1). The core concept of MK 4.0 recognizes that in neuroemergencies such as traumatic brain injury (TBI), subarachnoid hemorrhage (SAH), and ischemic stroke per example, cerebrovascular autoregulation (CA), the glymphatic system (GS) and cerebral compensatory reserve are key determinants of ICP regulation and patient outcomes [13, 18, 19]. Therefore, MK 4.0 underscores the importance of multimodal monitoring technologies in managing cerebral blood volume and pressure. It recognizes that cerebrovascular autoregulation (CA) failure, even in the absence of space-occupying lesions, can result in critical ICP elevations [20]. This insight is crucial for managing conditions that disrupt the brain’s natural mechanisms for regulating cerebral blood flow (CBF). The clinical application of MK 4.0 broadens treatment strategies beyond decompression or CSF drainage, incorporating multimodal monitoring. This approach enables personalized management aimed at optimizing cerebral perfusion within autoregulatory limits and glymphatic system function, preventing secondary brain injuries [21, 22].

## Cerebrovascular autoregulation role in MK 4.0

In contrast to other visceral organs, the brain is housed within a rigid bony structure and is surrounded by CSF and blood. The dynamic variations in their volumes directly influence ICP. As a result, cerebral perfusion in a healthy brain is not solely driven by the blood ejection pressure (arterial pressure, or ABP) within intracranial arteries. Instead, it is determined by the gradient between cerebral ABP and ICP [23]. State-of-the-art cerebral perfusion pressure (CPP) monitoring involves precisely quantifying the volume of blood reaching specific regions of brain tissue over a defined time interval. However, this approach relies exclusively on perfusion studies with limited temporal resolution and is not available at the bedside for critically ill patients.

As a result, extensive research has focused on identifying reliable surrogates for CPP estimation in both human and animal studies. However, in clinical practice and research on acute brain injuries, CPP is often calculated simply by subtracting intracranial pressure (ICP) from ABP, typically measured at the radial artery. This method overlooks potential inaccuracies and may lead to misleading conclusions [24–26]. In fact, several factors explain why the ABP-ICP difference does not accurately reflect CPP, as discussed below:

### Arterial blood pressure gradients from the heart to the brain and within the brain

CPP calculation remains highly variable across healthcare institutions, even among experienced clinicians managing brain-injured patients. This variability persists regardless of whether the setting is in high-income or low-to-middle-income countries, largely due to differences in how ABP is monitored [27]. It is essential to recognize that multiparameter monitors automatically calculate CPP using mean ABP and ICP inputs. However, the mean ABP at the heart may decrease by 10 to 15 mmHg by the time it reaches the skull base [28]. Ignoring this fact can lead clinicians to overestimate actual CPP. Additionally, cerebral arteries form an extensive branching network throughout brain tissue, causing a progressive decline in blood pressure within smaller arteries beyond the brain’s convexity—potentially reaching levels as low as half of the aortic pressure [29]. Neurocritical patients exhibit diverse pathophysiological conditions that are unevenly distributed across the brain, influenced by preexisting health conditions, injury severity, and disease progression [30]. Consequently, relying on CPP derived from global intracranial (ICP) and extracranial (ABP) measurements poses a significant risk of misguiding hemodynamic management.

### Disparity between counterforces ABP and ICP

**Fig. 2:**
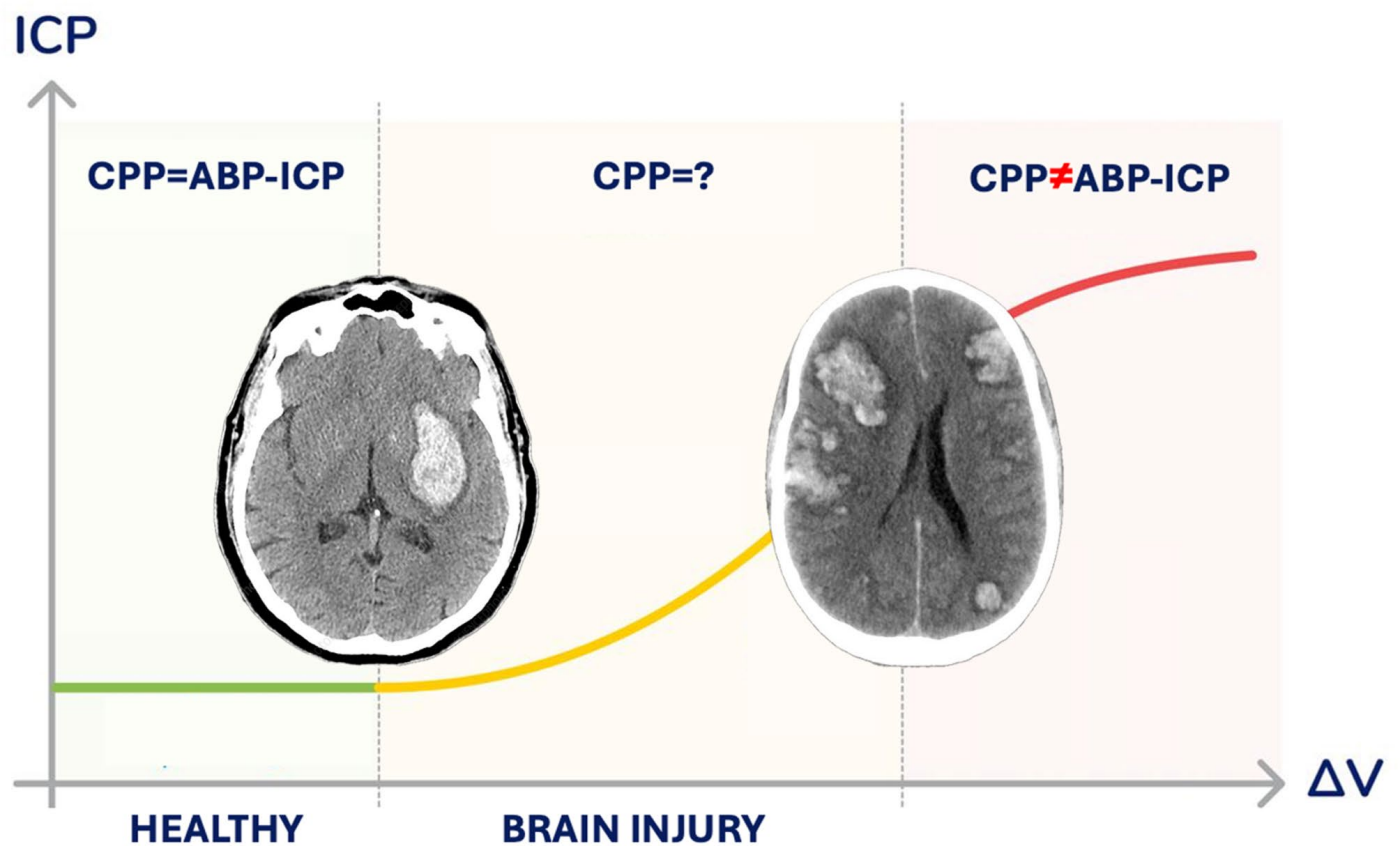
The ABP and ICP counterforces do not play an equal role in CPP compensation. After acute brain injuries with unknown CA impairment severity, as ICP raises, efforts on cerebral perfusion compensation by increasing ABP empirically may lead to further brain offense. ABP: arterial blood pressure, CA: cerebrovascular autoregulation, CPP: cerebral perfusion pressure, ICP: intracranial pressure

The contributions of ABP and ICP to CPP are not balanced. Due to the myogenic, metabolic, and autonomic properties of CA, CBF can effectively adapt to changes in ABP [31, 32]. However, in neurocritical patients, CA impairment varies and is directly proportional to the severity of intracranial hypertension [20, 33, 34]. Notably, studies have shown that in patients with severe brain injuries, even when ICP remains within normal ranges, a controlled mild elevation of approximately 5 mmHg can reduce cerebral blood velocity. This reduction persists longer than the step response observed following ABP changes [33]. Elevated ICP prolongs circulatory transit time [35, 36] and exerts a sustained influence on vessel transmural pressure (resistance-area product), which remains significantly elevated even after ICP reduction via decompressive craniectomy [33]. These findings indicate that different combinations of ABP and ICP can yield the same CPP calculation (e.g., 75 − 15 = 85 − 25), misrepresenting the true perfusion pressure within the brain’s microcirculation (CPP ≠ ABP - ICP), as illustrated in Fig. 2.

### Critical closing pressure and the lower limit of CA

The lower limit of CA —the threshold below which brain perfusion becomes insufficient to sustain neuronal activity— remains a topic of debate. While it is commonly accepted as 50 mmHg, a review of multiple studies suggests it may actually be closer to 70 mmHg [37]. In patients with severe brain injuries, CA is frequently impaired, reducing the brain’s ability to compensate for ABP fluctuations and narrowing the safe pressure range [20]. As a result, clinicians may inadvertently overlook the risk of brain hypoperfusion in normotensive patients, potentially leading to a complete absence of cerebral blood flow, particularly during the diastolic phase of the cardiac cycle. This phenomenon, where blood flow ceases despite a positive ABP, is known as critical closing pressure (CrCP). Research has shown that CrCP fluctuates with ICP variations, meaning that when intracranial hypertension is present, the threshold for zero cerebral blood flow increases [38].

### Cerebral perfusion pressure

The purpose of these arguments is to highlight the importance of personalized monitoring in unconscious neurocritical patients. Without an individualized approach, insufficient brain perfusion may occur even when systemic ABP remains within an acceptable range. Several studies in critically ill patients suggest that CPP may be a better predictor of outcomes than ICP alone [39]. As a result, one of the major goals in neurocritical care is identifying the optimal individualized CPP to guide patient management. Both excessively high and low CPP can be harmful, and the duration spent outside an individual’s autoregulatory limits may significantly impact outcomes [40–42]. The most studied and currently the only method for continuously calculating optimal CPP (CPPopt) relies on dedicated software (ICM+, Cambridge, UK). CPPopt is determined by identifying the ABP range that correlates best with ICP stability, measured through the pressure-reactivity index [43, 44]. While ABP-ICP correlation is not the ideal surrogate for cerebral perfusion, studies have shown that the longer patients remain outside the CPPopt range, the higher the mortality rate [45]. Unfortunately, this method requires both dedicated software and invasive ICP monitoring, significantly limiting its accessibility—especially in resource-limited settings and emergency rooms.

The next frontier in optimizing CPP management lies in developing methods that can deliver CPPopt widely in clinical settings. Among noninvasive approaches, transcranial Doppler (TCD) can be used to estimate CPP, offering a high positive predictive value for detecting reduced CPP—the condition clinicians are most concerned about [46]. However, TCD provides only intermittent measurements and requires specialized expertise, limiting its practicality. A major breakthrough would be the development of a reliable, point-of-care, noninvasive technique capable of continuously guiding clinicians in maintaining personalized cerebral perfusion targets.

## Intracranial dynamics materialization: the compartmental syndrome in MK 4.0

Given the evolution of the MK doctrine, it is necessary to redefine certain clinical situations that have traditionally been grouped under “intracranial hypertension”. The understanding that ICP is more than just a number has become increasingly clear [47–50]. A critical treatment decision point arises when intracranial compliance and brain oxygenation are compromised—even in patients with normal ICP values. Until now, even with the MK 3.0 advancements, clinical guidelines, consensus statements and protocols have recommended medical or surgical interventions exclusively in TBI and when ICP exceeds a predefined threshold (typically 20–22 mmHg). In mechanically ventilated patients, treatment escalation has traditionally been guided by a combination of ICP values, pupillary examination, and CT findings, often without considering other potential biomarkers [51].

However, as highlighted in MK 4.0, some patients exhibit exhausted intracranial compliance and brain hypoxia despite normal ICP values. These cases cannot be explained by systemic physiological factors, as identified through multi-monitoring parameters such as CBF, systemic hemodynamics, and systemic oxygenation. The focus of intracranial monitoring is shifting away from absolute ICP values toward ICP-derived parameters that have been shown to correlate more reliably with patient outcomes. These include: P2/P1 ratio and time-to-peak [36, 52], pulse shape index [53], RAP index [44] and ICP pulse amplitude [53, 54].

The concept of “Intracranial Compartment Syndrome (ICS)” has been proposed as a more precise approach to decision-making in TBI management [19], applying all instruments available to detect underlying additional brain injuries. This concept integrates early invasive and/or noninvasive intracranial compliance monitoring and invasive and/or noninvasive brain oxygenation techniques, across emergency, surgical, and ICU settings. ICS allows for the early identification of high-risk patients who require aggressive treatment independently of ICP values [19, 47]. If left unrecognized, this progressive syndrome often leads to a cascade of high-mortality events, including severe intracranial hypertension, brain tissue hypoxia and brain herniation. By recognizing ICS as a distinct entity, clinicians can implement earlier and more targeted interventions, potentially reducing morbidity and mortality in acute brain injury patients.

Before recognizing this missing concept, treatment efforts were traditionally intensified only for patients who were already critically ill or had a high burden of brain injury. This approach may have contributed to the biased perception that treatment is futile for moderate to severe traumatic brain injury patients worldwide [55–57]. Future research must identify non-traditional factors contributing to the early exhaustion of intracranial compliance and how this deterioration can lead to brain hypoxia—even before the full onset of traditional intracranial hypertension syndrome.

Invasive ICP monitoring remains as a cornerstone in neurocritical care, however, an ICP static threshold-based model does not fit in MK 4.0. In this context, changes in ICP pulse morphology represent a critical area of focus [58–60]. ICP waveforms reflect the relationship between pressure and intracranial compliance, key markers of early neurological deterioration as emphasized in Monro-Kellie 4.0 [61, 62]. While invasive ICP monitors can assist clinicians in capturing this information, current interpretations of ICP wave morphology remain largely subjective. This is because existing invasive systems do not provide quantitative metrics derived from the deformation of ICP pulse shapes. Some manufacturers of invasive ICP monitors have acknowledged this limitation and have announced plans to include such parameters alongside traditional ICP values in the near future [63]. A groundbreaking discovery revealed that micrometric cranial movements following each heartbeat [8] can reproduce ICP waveforms, enabling the development of a noninvasive method for monitoring intracranial compliance with multiple clinical applications [36, 52, 64, 65]. This innovation holds the potential to make ICP monitoring as accessible and routinely measured as any other vital sign [66].

Varied conditions such as thicker cranial vault bones, untreated cranial stenosis (reducing skull capacity), abnormal venous circulation (including venous thrombosis and anatomical drainage variations), and impaired glymphatic circulation (shift edema) can contribute to the early stages of ICS following acute brain injury [67–69]. These factors are often overlooked in routine clinical assessments or standard imaging during initial patient evaluations.

The early integration of noninvasive neuromonitoring systems, since the emergency department and early integration of invasive systems at the ICU provides valuable insights into how these conditions impact brain perfusion, leading to broader global adoption [27]. This new approach enhances patient triage and enables the early identification of red flags, fostering a more personalized and precise treatment strategy under the MK 4.0 framework. The recent publication of the B-ICONIC consensus on noninvasive ICP monitoring, alongside earlier efforts to evaluate the role of invasive systems (the SIBICC protocols), represents an important initial step toward establishing a conceptual foundation for validating the ICS in future clinical research [22, 70].

## The glymphatic system relevance in MK 4.0

When analyzing the MK doctrine in relation to the components of the cranial cavity and their influence on ICP, the interstitial space (IS) has traditionally been considered a minor player. This is likely due to its small volume fraction (approximately 3.5%) within the cranial compartment [71]. However, recent pathophysiological advancements have reshaped this perspective, highlighting the IS and its components as key contributors to intracranial dynamics [72–74]. Unlike most tissues, the brain parenchyma lacks a conventional lymphatic system, except for the meninges, which connect to extracranial lymphatic vessels and nodes, primarily in the neck [75]. As a result, brain waste clearance has traditionally been attributed to intracellular degradation or elimination through the bloodstream via the blood-brain barrier, relying on slow diffusion or active transport mechanisms [76].

The GS function requires significant energy expenditure [72–74] and is primarily driven by arterial pulsatility (para-arterial inflow) [77], CSF pressure gradients [78], respiratory cycles [79, 80] and the vasomotor tone [81]. Fluid exchange between the IS and CSF occurs in the terminal portion of the Virchow-Robin space via diffusion, where the absence of a fibrous matrix provides minimal resistance. The transport of fluids and solutes across the cerebral IS to the perivascular venous space is driven by convective flow, where astrocytes AQP4 plays a critical role facilitating the movement of water across cell membranes [74]. Once in the venous space, the collected interstitial fluid exits the brain and drains into the cervical lymphatic system. This mechanism promotes the clearance of metabolic waste from cellular activity, modulates neuronal excitability by indirectly affecting ionic balance and neurotransmitters homeostasis.

**Fig. 3:**
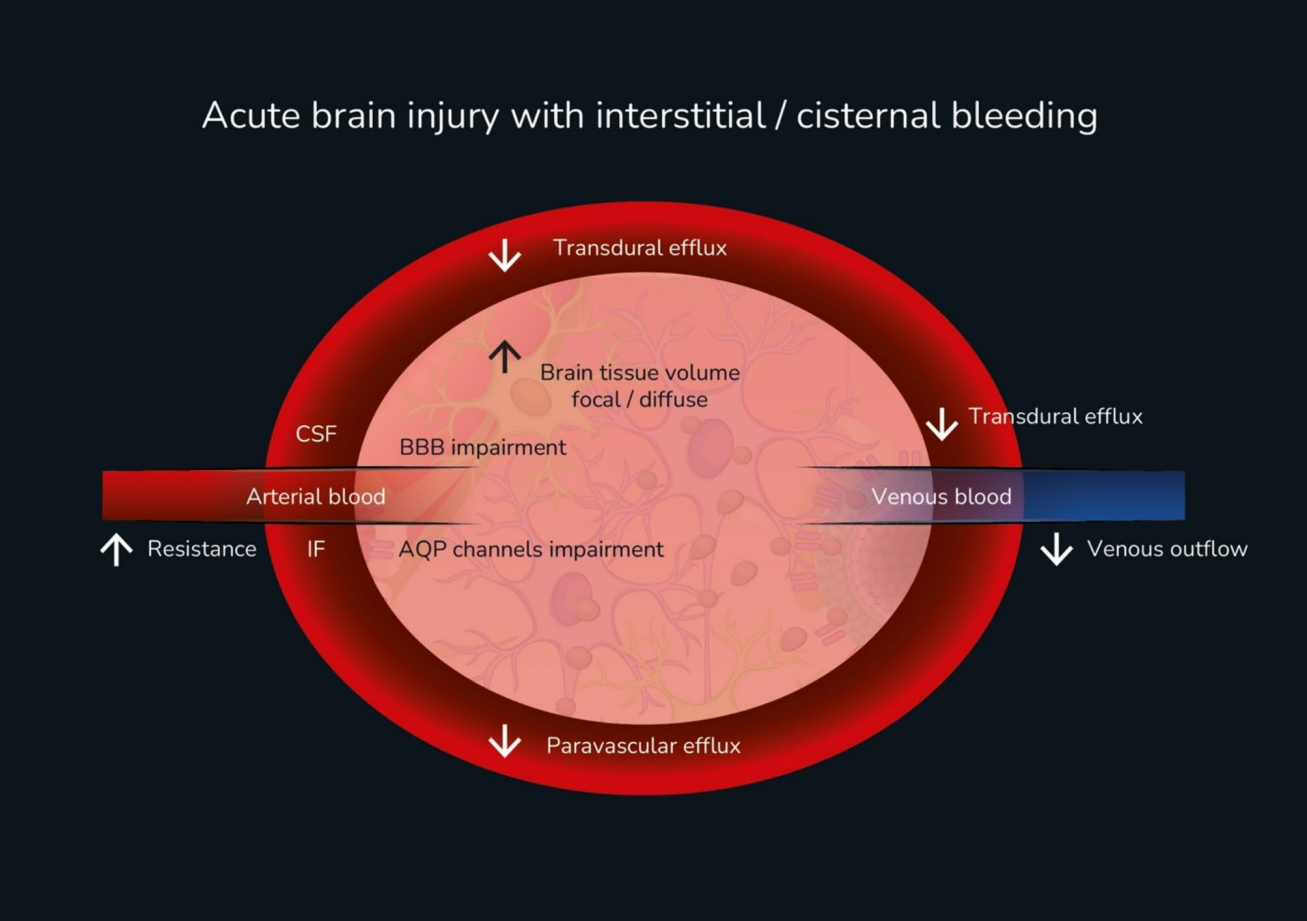
Mechanisms for glymphatic system impairment in acute brain injuries. Interstitial bleedings, intracranial hypertension and decompressive craniectomy contribute for increasing vascular resistance and reducing arterial pulsation, AQP4 dysfunction, accumulation of cerebral metabolism debris, neurotoxins and citoquines. These multiple mechanisms reinforce the need for CSF drains when bleedings reach the cisterns and Virchow-Robin spaces. AQP4: aquaporin 4, BBB: brain-blood barrier, CSF: cerebrospinal fluid

GS dysfunction has been linked to multiple mechanisms of cellular and tissue damage (Fig. 3) [82]. As a consequence of injury response, neuroinflammation and immune cascades (TNF-α, interleukines-1β, 6,8,10, IFN-γ and matrix metalloproteinases MMP2-MMP9) in the IS contribute to brain edema formation and progression [73, 83]. Regarding the GS, the most significant concept considered in Monro-Kellie 4.0 is the shift edema hypothesis. According to this theory, patients with traumatic or spontaneous subarachnoid hemorrhage may develop secondary brain edema due to GS obstruction [84, 85]. By removing injured red blood cells from the subarachnoid space, cisternostomy may help restore glymphatic function, leading to better ICP regulation, enhanced clearance of neurotoxic metabolites, and reduction of cerebral edema. Emerging evidence suggests that effective cisternal drainage improves perivascular flow, supports brain fluid homeostasis and may facilitate neurological recovery [82, 86, 87].

Although large prospective trials are still lacking, several additional therapeutic strategies aimed at improving GS function show promising clinical potential [74, 83, 88, 89]. Some approaches include optimizing sedation and analgesia levels, modulating sleep patterns, reducing catecholamine activity, targeting AQP4 and associated receptors (e.g., SUR1) and creating osmotic gradients to stimulate convective flow [90–92].

## Emergency department evaluation of the MK 4.0 paradigm shift

In traditional approaches, dysfunction of the principal MK component (ICP) was confined to the ICU by invasive measurement systems, regularly available in high resourced settings. With this new approach the process can start earlier, identifying intracranial compliance impairment through at least two different noninvasive methods—such as pupillometry, noninvasive ICP waveform analysis, optic nerve ultrasound, or transcranial Doppler—can provide the clinical team with valuable insights into the pathophysiological processes occurring inside the skull. In regions without access to CT scans, this assessment helps determine the urgency of patient transfer. In centers with CT availability, combining imaging of the primary injury with intracranial compliance evaluation enhances decision-making, enabling more precise and timely surgical interventions [70].

**Fig. 4:**
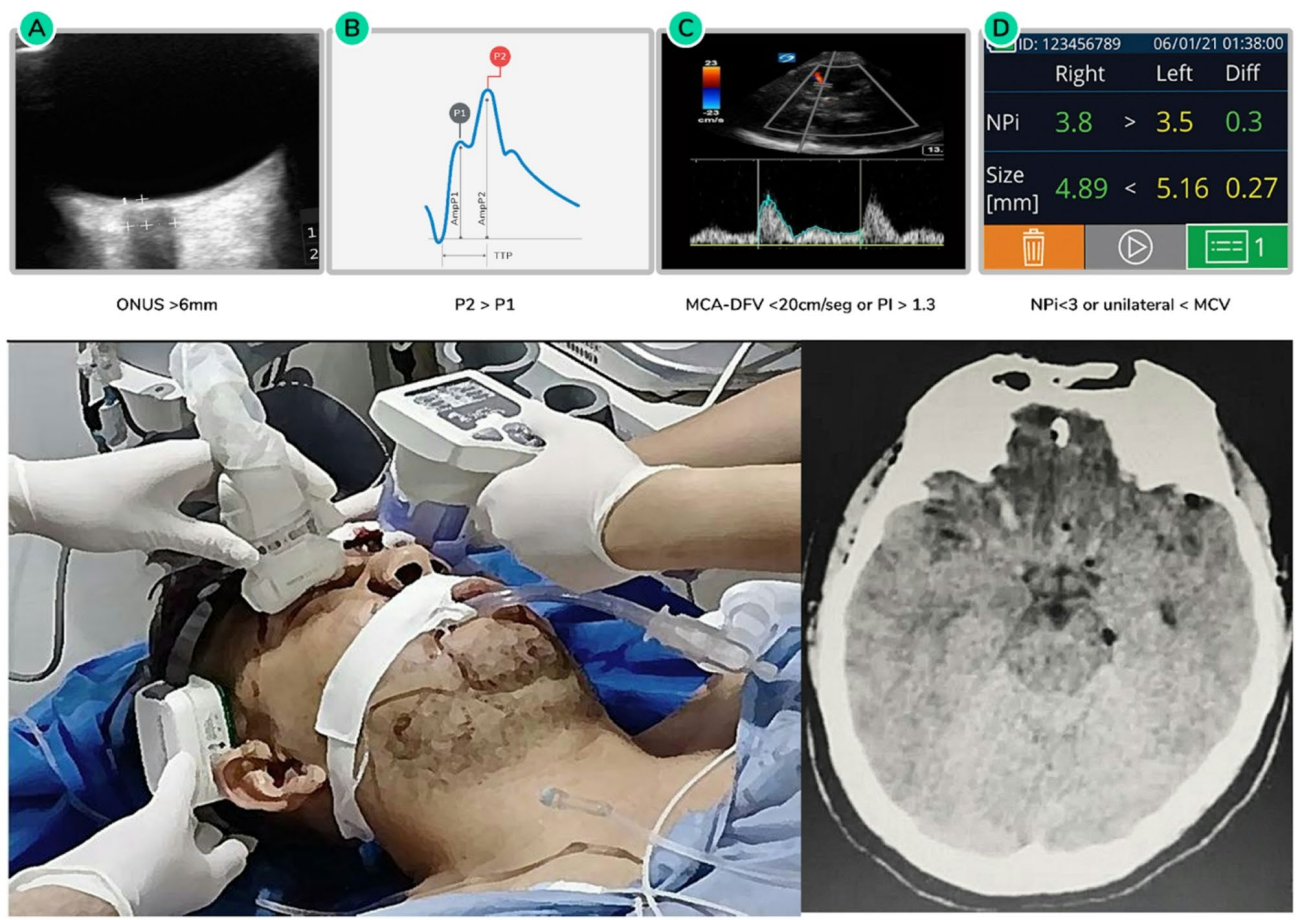
Noninvasive evaluation of intracranial compliance (ICC) in the emergency setting within the MK 4.0 paradigm shift. A severe TBI patient presenting a Marshal II injury at admission. ONSD ultrasound, noninvasive ICP waveform analysis and TCCD (A, B and C respectively) performed in the ER were indicative of ICC impairment, whereas NPi (D) was still favorable. Noninvasive assessment of an impending neurological deterioration. ER: emergency room, ICP: intracranial pressure, ONSD optic nerve sheath diameter, TCCD: transcranial color coded duplex, TBI: traumatic brain injury

Figure 4 presents a case of TBI involving a depressed frontal skull fracture with a small frontal-basal contusion, minor pneumocephalus, and a slight midline shift of less than 5 mm. Based on current protocols, the primary injury does not require immediate surgery. However, at least three noninvasive monitoring systems detected intracranial compliance abnormalities. Despite these findings, the Neurological Pupil Index (NPi) remains normal on both sides, indicating that the patient is not yet experiencing brain herniation. Given the abnormal compliance findings, urgent continuous monitoring of brain compliance and oxygenation by invasive or noninvasive systems is necessary. Additionally, autoregulation status and electrical activity should be frequently reassessed in the ICU setting.

If invasive monitoring is unavailable in an ICU, then, noninvasive assessments can be performed at least three to four times per day to track trends [70]. If deterioration occurs despite medical treatment, early surgical intervention should be considered to improve compliance on the most affected side. If invasive monitoring is available, then continuous measurement will allow also identification of compliance or oxygenation impairments. As discussed earlier, CSF drainage methods—such as external ventricular drainage (EVD), external lumbar drainage (ELD), or cisternostomy—play a crucial role beyond just reducing intracranial volume. Ultimately, cranial decompression should be considered a last resort if the patient does not improve with prior interventions.

## Limitations of the MK 4.0 approach

Acute brain injuries, particularly severe TBI, are highly heterogeneous conditions, making the design and execution of interventional clinical trials challenging. It remains to be proven whether earlier recognition of secondary brain injuries through this approach can improve patient outcomes. The association of invasive and noninvasive neuromonitoring model proposed by the B-ICONIC consensus has yet to undergo prospective validation. Additionally, while the pathophysiological insights presented in this manuscript highlight the importance of glymphatic function and CA, definitive strategies for restoring glymphatic function or determining optimal CA ranges are still under development.

## Conclusion

The Monro-Kellie doctrine must evolve to reflect the latest advancements in cerebral physiology and technology. The MK 4.0 update integrates recent discoveries on cerebrovascular autoregulation and the glymphatic system, recognizing them as key contributors to intracranial dynamics and intracranial compliance. By redefining these principles, the doctrine now provides a more precise framework for understanding and managing neuroemergencies, paving the way for more effective, personalized treatment strategies.

## Data Availability

No datasets were generated or analysed during the current study.

## Abbreviations

ABI: Acute brain injury
ABP: Arterial blood pressure
CA: Cerebrovascular autoregulation
TBI: traumatic brain injury
ICP: Intracranial pressure
ICPW: Intracranial pressure waveform
IF: Interstitial fluid
IS: interstitial space
CSF: Cerebrospinal fluid
GS: Glymphatic system
CT: Computed tomography
IH: Intracranial hypertension
ICCS: Intracranial compartmental syndrome
CBV: Cerebral blood volume
ICC: Intracranial compliance
CPP: Cerebral perfusion pressure
MAP: Mean arterial pressure
TCD: Transcranial doppler
NPV: Negative predictive value
